# Hematopoietic-Specific Deletion of Foxo1 Promotes NK Cell Specification and Proliferation

**DOI:** 10.3389/fimmu.2019.01016

**Published:** 2019-05-08

**Authors:** Pei Huang, Fangjie Wang, Yao Yang, Wenjing Lai, Meng Meng, Shuting Wu, Hongyan Peng, Lili Wang, Rixing Zhan, Saber Imani, Jianhua Yu, Bingbo Chen, Xiaohui Li, Youcai Deng

**Affiliations:** ^1^Institute of Materia Medica, College of Pharmacy, Army Medical University (Third Military Medical University), Chongqing, China; ^2^Hunan Children's Hospital, Hunan Children's Research Institute (HCRI), University of South China, Changsha, China; ^3^Southwest Hospital, Institute of Burn Research, Army Medical University (Third Military Medical University), Chongqing, China; ^4^Department of Oncology, The Affiliated Hospital of Southwest Medical University, Luzhou, China; ^5^Department of Hematology and Hematopoietic Cell Transplantation, City of Hope National Medical Center, Duarte, CA, United States; ^6^Laboratory Animal Center, Army Medical University (Third Military Medical University), Chongqing, China

**Keywords:** Foxo1, natural killer cell, NK specification, NK proliferation, cell cycle

## Abstract

We previously reported that deletion of Foxo1, *via Ncr1*-iCre mice from the expression of NKp46 onward, led to enhanced natural killer (NK) cell maturation and effector function. In this model, however, the role of Foxo1 in regulating NK cell specification and early development remains exclusive. Herein, we utilized a murine model of hematopoietic-specific deletion of Foxo1 before lymphoid specification, by crossing mice carrying floxed *Foxo1* alleles (*Foxo1*^fl/fl^) with *Vav1*-iCre mice, to revisit the role of Foxo1 on NK cell specification and early development. The data showed that hematopoietic-specific deletion of Foxo1 resulted in increased proportion and numbers of common lymphoid progenitors (CLP) (Lin^−^CD127^+^c-Kit^+^Sca-1^+^), pre-pro NK b cells (Lin^−^Sca-1^+^c-Kit^−^CD135^−^CD127^+^), as well as committed Lin^−^CD122^+^ cells and CD3^−^CD19^−^NKp46^+^ NK cells in bone marrow. Hematopoietic-specific deletion of Foxo1 also promoted NK cells proliferation in a cell-intrinsic manner, indicated by increased Ki-67 expression and more expansion of NK cell after *ex vivo* stimulation with IL-15. The reason for Foxo1 suppressing NK cell proliferation might be its direct transcription of the cell-cycle inhibitory genes, such as *p21*^cip1^, *p27*^kip1^, *p130, Gadd45a*, and *Ccng2* (*cyclin G2*) in NK cells, supported by the evidence of decreased mRNA expression of *p21*^cip1^, *p27*^kip1^, *p130, Gadd45a*, and *Ccng2* in Foxo1-deficient NK cells and direct binding of Foxo1 on their promoter region. Furthermore, hematopoietic-specific deletion of Foxo1 resulted in increased ratio of mature NK subsets, such as CD11b^+^CD27^−^ and CD43^+^KLRG1^+^ NK cells, but decreased ratio of immature NK subsets, such as CD27^+^CD11b^−^ and CD27^+^CD11b^+^ NK cells, consistent with the findings in the murine model of *Ncr1*-iCre mediated Foxo1 deletion. Conclusively, Foxo1 not only acts as a negative checkpoint on NK cell maturation, but also represses NK cell specification and proliferation. The relative higher expression of Foxo1 in CLP and early NK precursors may also contribute to the later NK cell proliferation and responsiveness, which warranties another separate study in the future.

## Introduction

Natural killer (NK) cells are a distinct subset of group 1 innate lymphoid cells with a crucial role in innate immunity (1). Cytokine secretion and granule-mediated cytotoxicity are the two main effector functions of NK cells (2). Their cytotoxic function is critical to many immune responses, including tumor immunosurveillance and elimination of viral infection (3).

NK cells develop mainly in the bone marrow (BM) from hematopoietic stem cells (HSCs) (4). Murine NK cells derive from common lymphoid progenitors (CLP) through three major steps defined as: CD122^+^NK1.1^−^DX5^−^ NK cell precursors (NKps), CD122^+^NK1.1^+^DX5^−^ immature NK (iNK) cells and CD122^+^NK1.1^+^DX5^+^ mature NK (mNK) cells (4). During the late stage of maturation, NK cells gradually upregulate CD11b expression, downregulate CD27 expression, and finally obtain co-expression of CD43 and KLRG1 (5–8). Previous studies have revealed several transcriptional factors that are positive required for each stage of NK cell development, with little known about the negative ones (9).

The Foxo family is critical to many aspects of cellular physiology (10). In this family, we previously found that Foxo1 is the most highly expressed, compared with Foxo3 and 4 and 6, in NK cells and revealed that Foxo1 negatively regulated NK cell maturation and effector function (11). However, in that study, we crossed *Ncr1*-iCre mice with mice harboring floxed Foxo1 alleles (Foxo1^fl/fl^) to specifically delete Foxo1 from the expression of NKp46 onward in NK cells, which meant that Foxo1 deletion only occurred in NK and NK cells. Therefore, there remains an interesting question that what's the role of Foxo1 in NK specification and early development, as the protein level of Foxo1 was much higher in NKPs and iNKs as compared to that in mNK cells (11). In this current study, we used *Vav1*-iCre mice (12) to cross with *Foxo1*^fl/fl^ mice to generate hematopoietic-specific Foxo1-deleted mice (*Foxo1*^fl/fl^*/Vav1*-iCre^+^), which could delete Foxo1 expression before lymphoid specification, including HSCs, CLPs, NKp, mNK cells, and also other lymphocytes. In this model, we found that hematopoietic-specific deletion of Foxo1 resulted in increased ratio and cell numbers of CLPs, pre-pro NK, and NK cells, together with enhanced NK cell proliferation, which was inconsistent with the findings by *Ncr1*-iCre deletion model. Hematopoietic-specific deletion of Foxo1 showed a similar effect on NK cell maturation in the murine model of *Ncr1*-iCre-mediated Foxo1 deletion.

## Materials and Methods

### Animals

FVB/129S6 *Foxo1*^fl/fl^ mice (13) and C57BL/6 *Vav1*-iCre (12) mice were purchased from the Jackson Laboratory. *Foxo1*^fl/fl^ mice were crossed with *Vav1*-iCre mice to generate F1 Foxo1^fl/+^/*Vav1*-iCre mice. The F1 progeny was further backcrossed to FVB/129S6 *Foxo1*^fl/fl^ mice for at least 12 generations to obtain littermate control, *Foxo1*^fl/fl^, and *Foxo1*^fl/fl^/*Vav1*-iCre (named as Foxo1^Δ/Δ^) mice. All mice were bred and maintained in specific pathogen-free conditions in the Experimental Animal Center of the Army Medical University. Seven to eight weeks old mice were used in our experiments. All animal related procedures and protocol were approved by the Animal Ethics Committee of the Army Medical University, and followed the guidelines of the Institutional Animal Care and Use Committees of the Third Military Medical University (Chongqing, China).

### Flow Cytometry

Flow cytometry was performed as described previously (11, 14). In details, single cells were isolated from mouse BM, spleen, and periphery lymph nodes (pLN). If necessary, BM cells were counted by Fuchs-Rosenthal Counting Chamber. Briefly, to analyze the surface marker, cells were blocked with anti-CD16/CD32 antibodies (eBioscience, San Diego, CA) and then stained with surface marker antibodies and washed by PBS. To analyze the intracellular proteins, after staining with surface markers, cells were fixed and permeabilized with Foxp3/Transcription Factor Staining Buffer Kit following the manufacturer's protocols (eBioscience, San Diego, CA).

Antibodies used were as followings: anti-Foxo1 (C29H4) was from Cell Signaling Technology (Boston, MA); anti-NKp46 (29A1.4), anti-CD19 (6D5), anti-CD3 (17A2), anti-Gr1 (RB6-8C5), anti-Ter119 (Ter119), anti-CD4 (GK1.5), anti-CD8 (53-6.7), anti-c-Kit (2B8), anti-Sca-1 (D7), anti-CD127 (A7R34), and anti-CD49b (DX5) were from Biolegend (San Diego, CA); anti-CD27 (LG.3A10), anti-CD11b (M1/70), anti-B220 (RA3-6B2), and anti-CD122 (TM-β1) were form BD Biosciences (San Diego, CA); anti-CD43 (S7), anti-KLRG1 (2F1), anti-CD135(A2F10), anti-Ki-67 (SolA15), and anti-T-bet (4B10) were from eBioscience (San Diego, CA).

### Apoptosis Assay

Fresh BM and spleen cells were performed with apoptosis assessment using an Annexin V apoptosis detection kit (BD Biosciences, San Diego, CA), according to the manufacturer's instructions and also as previously described in detail (14).

### NK Cell Purification and Quantitative RT-PCR Analysis

Single splenic cells from littermate control and Foxo1^Δ/Δ^ mice were stained with anti-CD3, anti-CD19, and anti-NKp46 antibodies. Then, CD3^−^CD19^−^ NKp46^+^ NK cells were sorted by FACSAria™ III (BD Biosciences, San Diego, CA).

Total RNA of 400,000 NK cells were purified and then subjected to reverse transcription and quantitative real-time PCR (qRT-PCR) reaction as previously described (14). *Hprt1* was used as internal control and relative mRNA expression were calculated by normalizing the relative cycle threshold value to the control group. The primer pairs used were listed in Table 1.

**Table 1:** Quantitative real time PCR for cell-cycle repressor genes.

**Gene symbol**	**Forward (5′-3′)**	**Reverse (5′-3′)**
*p21*^CIP1^	CCTGGTGATGTCCGACCTG	CCATGAGCGCATCGCAATC
*p27*^kip1^	TCAAACGTGAGAGTGTCTAACG	CCGGGCCGAAGAGATTTCTG
*p130*	AACTTCCCCATGATTAGCGATG	GGTTAGAACACTGAAGGGCATTT
*Gadd45a*	AGACCGAAAGGATGGACACG	GTACACGCCGACCGTAATG
*cyclinD1*	GCGTACCCTGACACCAATCTC	CTCCTCTTCGCACTTCTGCTC
*cyclinD2*	GAGTGGGAACTGGTAGTGTTG	GCACAGAGCGATGAAGGTC
*Ccng2*	GGGGTTCAGCTTTTCGGATTG	AGATCAGCCCTTTTTCCCGAG
*Hprt1*	GCTGGTGAAAAGGACCTCT	CACAGGACTAGAACACCTGC

### Chromatin Immunoprecipitation (ChIP) Assay

ChIP assay was performed with EZ-Magna ChIP™ A/G Chromatin Immunoprecipitation Kit following the manufacturer's instructions (Merck Millipore, Darmstadt, Germany) and also as described previously (11). Briefly, 4 × 10^6^ mouse splenic NK cells were sorted with purity over 95%. To precipitate cross-linked protein-DNA complex for further PCR reaction, an equal amount (5 μg) of anti-Foxo1 antibodies (C29H4, Cell Signaling Technology, Boston, MA) and control IgG antibodies (Merck Millipore, Darmstadt, Germany) were added. The PCR primers spanning the different Foxo1 binding sites on the promotor region of each cell repressor genes were listed in Table 2.

**Table 2:** Primers used for Foxo1 ChIP.

**Gene symbol**	**Foxo1 binding site**	**Forward (5′-3′)**	**Reverse (5′-3′)**
*p21*^CIP1^	−1722~-1712	TCCGTTCAAACTAAGACTCCA	TAGCGCTTGCCTAACATGTAT
*p27*^kip1^	−1386~-1376	ACACACATCCTGGCAAAGA	CCTGTCGTATCTCAGAGTTCA
*p27*^kip1^	−1036~-1026	TAGATGTTGGTAATACCGTGG	CGCCTTTATACCCTTATGTTC
*p130*	−640~-630	CGTATCTAGGAGCAGTTATGTG	CCCACAGGTCAATCCAACAG
*p130*	−356~-346	CGTCCTGCTTTAACCTCCGT	CCTTTACACACATCCTCACCTA
*Gadd45a*	−1061~-1051	TAGGTTCAGGCAATGCTTTT	TTCACCTTTGAGATAATAGCA
*Gadd45a*	−432~-422	CAGGGCACCAAAAGACTACTA	ATCTTTTGATTGTCGTTTCGT
*Ccng2*	−1519~-1509	CTACCTTTGGCCATTGGAAC	ATGCTTGTAAACTCGCACAG

### *In vitro* NK Cell Culture

Purified NK cells (>95%) were cultured in RPMI1640 (10% FBS and Penicillin-Streptomycin) (Gibco, Invitrogen Australia Pty Ltd, Victoria, Australia) with 50 or 100 ng/ml murine IL-15 (Biolegend, San Diego, CA). After 5 days of culture, cell numbers were counted by Fuchs-Rosenthal Counting Chamber and NK cell apoptosis was performed as described previously (14).

### Statistical Analysis

Unpaired Student's *t*-test was utilized to compare two independent groups. Generalized linear models were used in the randomized block design with litters as the block factor. All reported *p*-values < 0.05 was considered statistically significant.

## Results

### Hematopoietic-Specific Deletion of Foxo1 Leads to Increased Ratio and Numbers of CLP, Pre-pro NK, and Committed NK Cells

**Figure 1:**
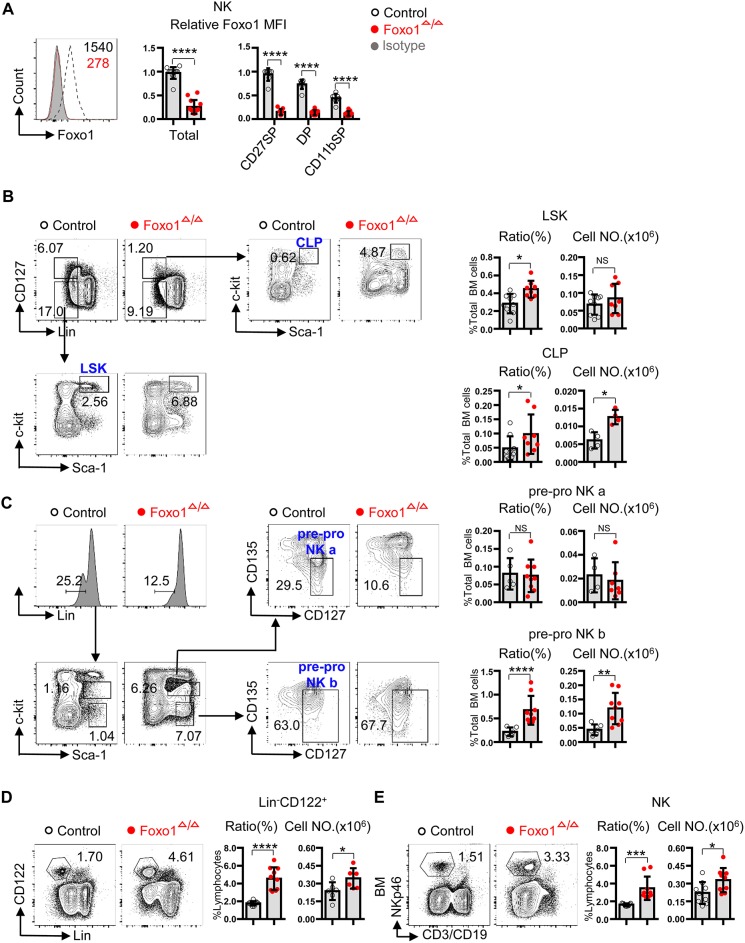
Hematopoietic-specific deletion of Foxo1 resulted in increased proportion and quantity of CLP, pre-pro NK, and NK cells. **(A)** Intracellular flow cytometric analysis of Foxo1 expression (mean fluorescence intensity (MFI) relative to littermate control) in splenic NK cells (CD3^−^CD19^−^NKp46^+^) from control vs. Foxo1^Δ/Δ^ mice. **(B)** Frequency and enumeration of LSK (Lin^−^CD127^−^Sca-1^+^c-Kit^+^) and CLP (Lin^−^CD127^+^Sca-1^+^c-Kit^+^) in the bone marrow (BM) from control vs. Foxo1^Δ/Δ^ mice. Lin: CD3, CD19, B220, CD4, CD8, CD11b, NKp46, Gr1, and Ter119. **(C)** Frequency and enumeration of pre-pro NK a (Lin^−^Sca-1^+^c-Kit^int^CD135^−^CD127^+^) and pre-pro NK b (Lin^−^Sca-1^+^c-Kit^−^CD135^−^CD127^+^) in the BM from control vs. Foxo1^Δ/Δ^ mice. Lin: CD3, CD19, B220, Gr1, Ter119, CD4, CD8, CD11b, and NKp46. **(D)** Frequency and enumeration of Lin^−^CD122^+^ cell in the BM from control vs. Foxo1^Δ/Δ^ mice. Lin: CD3, CD19, Gr1, and Ter119. **(E)** Frequency and enumeration of NK cells (CD3^−^CD19^−^NKp46^+^) in the BM from control vs. Foxo1^Δ/Δ^ mice. Ratio of LSK, CLP, pre-pro NK a and pre-pro NK b cells are relative to total BM cells **(A–C)**, whereas ratio of Lin^−^CD122^+^ and CD3^−^CD19^−^NKp46^+^NK cells are relative to total lymphocytes. Each dot represents one mouse. At least 3 littermates were included for **(A–E)** (Error bars indicate SD; ^*^p < 0.05, ^**^p < 0.01, ^***^p < 0.001, and ^****^p < 0.0001, control vs. Foxo1^Δ/Δ^ mice; unpaired Student's *t*-test with generalized linear models).

To interrogate the role of Foxo1 in the specification and early stages of NK cell development, we crossed *Vav1*-iCre (12) with *Foxo1*^fl/fl^ (13) mice to acquire littermate control (*Vav1*-iCre^−^*Foxo1*^fl/fl^) and hematopoietic-specific deleted Foxo1 (*Vav1*-iCre^+^*Foxo1*^fl/fl^, named as Foxo1^Δ/Δ^) mice. Intracellular flow cytometry analysis exhibited the efficient deletion of Foxo1 in splenic CD3^−^CD19^−^NKp46^+^ total NK cells, as well as each subset based on the expression of CD27 and CD11b (15, 16) (Figure 1A). As Foxo1 could be deleted before lymphoid specification in *Vav1*-iCre mice (12), we firstly determined the ratio and cell number of LSK, CLP as well as early NK progenitors in Foxo1^Δ/Δ^ mice. The data showed that Foxo1^Δ/Δ^ mice showed increased proportion and quantity of CLP (Lin^−^CD127^+^-c-Kit^+^Sca-1^+^) (17), without any significance in numbers of LSK (Lin^−^CD127^−^c-Kit^+^Sca-1^+^) (17) in the BM of Foxo1^Δ/Δ^ mice (Figure 1B). Due to the lack of CD244 expression, which is a critical marker for gating pre-NKPs (lineage^−^CD27^+^c-Kit^−^CD135^−^CD244.2^+^CD127^+^CD122^−^) and rNKPs (lineage^−^CD27^+^c-Kit^−^CD135^−^CD244.2^+^CD127^+^CD122^+^) in C57/BL6 mouse (18–20), we used another strategy to explore the role of Foxo1 on early NK cell progenitors, pre-pro NK cells (Lin^−^Sca-1^+^c-Kit^int/−^CD135^−^CD127^+^) (21). Due to the protein levels of c-Kit, pre-pro NK cells could be further divided into two subsets: pre-pro NK a (Lin^−^Sca-1^+^c-Kit^int^CD135^−^CD127^+^) and pre-pro NK b (Lin^−^Sca-1^+^c-Kit^−^CD135^−^CD127^+^) (21). Our data showed that pre-pro NK b, but not the pre-pro NK a, exhibited robust increase in both ratio and cell numbers in the BM of Foxo1^Δ/Δ^ mice (Figure 1C). As refer to the committed NK cells after acquiring the expression of CD122, Foxo1^Δ/Δ^ mice showed increased proportion and quantity of Lin^−^CD122^+^ cells and CD3^−^CD19^−^NKp46^+^ NK cells in the BM (Figures 1D,E).

As Foxo1 could be deleted in HSC, CLP as well as other lymphocytes in *Vav1*-iCre mice (12), we also explored the role of Foxo1 on the proportions and cell numbers of B and T cells in Foxo1^Δ/Δ^ mice. The ratio and cell numbers of B cells and CD3^+^ T cells were tremendously reduced in Foxo1^Δ/Δ^ mice (Supplemental Figures 1A,B), consistent with previous published data (22). The total cell numbers, but not the ratio, of CD4^+^ T cells and CD8^+^ T cells were all decreased in the BM (Supplemental Figure 1B). Although the significantly reduced cell numbers of T and B cells in Foxo1^Δ/Δ^ mice, both the CD4^+^ and CD8^+^ T cells showed an obvious enhanced activation phenotype, as indicated by the expression of CD44, CD62L and CD69 (23). The ratio of CD44^+^CD62L^−^ and CD69^+^ activated CD4^+^ T cell were significantly increased in Foxo1^Δ/Δ^ mice (Supplemental Figure 1C).

Collectively, these data suggested that Foxo1 negatively controlled NK specification and early development, contrast to its positive role on B and T cell development. All these findings were inconsistent with the model of *Ncr1*-iCre-mediated Foxo1 deletion in our previous study (11).

### Hematopoietic-Specific Deletion of Foxo1 Results in Enhanced NK Cell Proliferation

**Figure 2:**
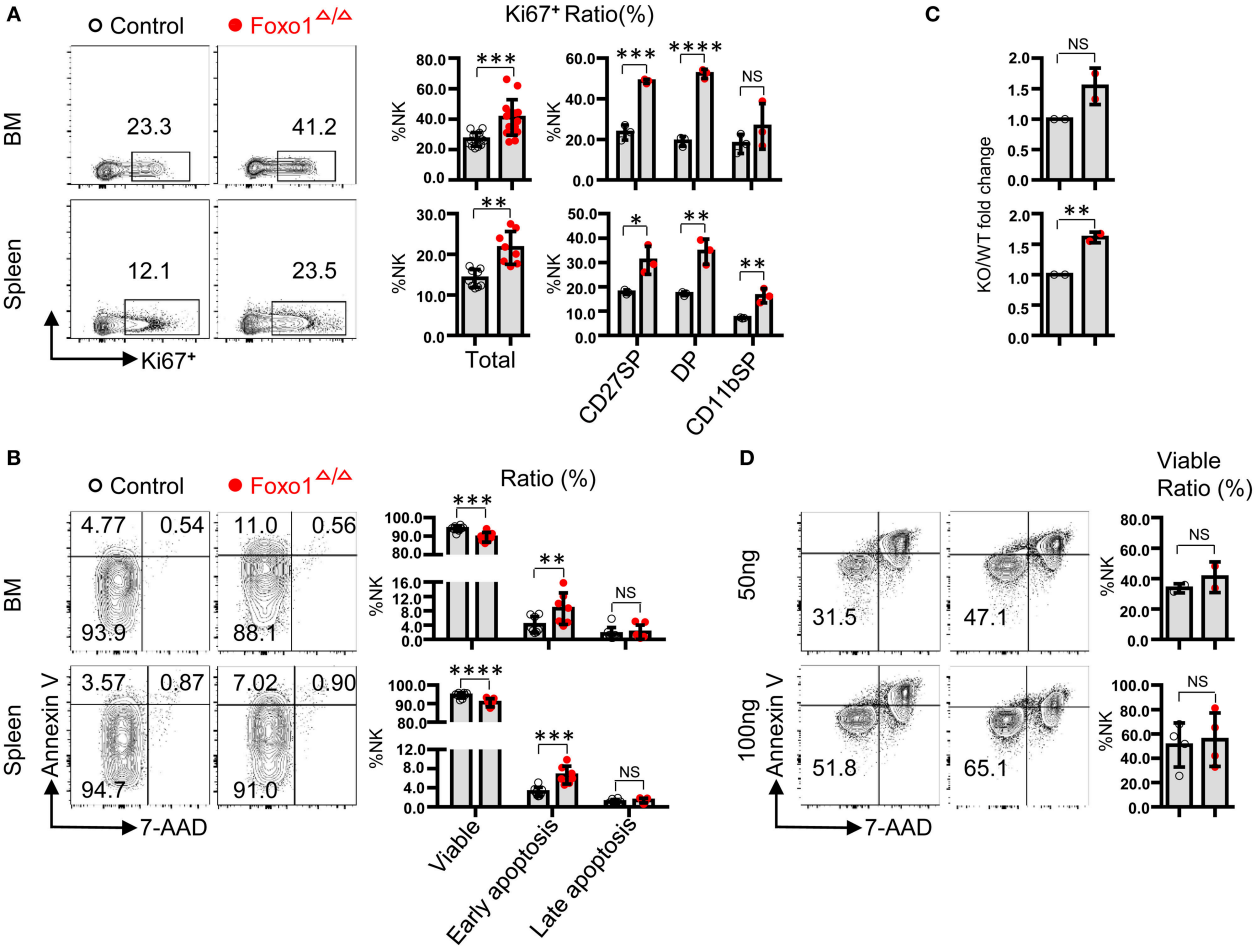
Hematopoietic-specific deletion of Foxo1 leads to increased proliferation of NK cells. **(A)** Intracellular flow cytometric analysis of Ki-67^+^ cells in total NK cells (CD3^−^CD19^−^ NKp46^+^) and within indicated subpopulations in both the BM and spleen from control vs. Foxo1^Δ/Δ^ mice. **(B)** Flow cytometric analysis of the apoptosis of NK cells (CD3^−^CD19^−^ NKp46^+^) in both the BM and spleen from control vs. Foxo1^Δ/Δ^ mice. Viable: Annexin V^−^7-AAD^−^subpopulation; early apoptosis: Annexin V^+^7-AAD^−^subpopulation; late apoptosis: Annexin V^+^7-AAD^+^subpopulation. **(C,D)** Enumeration **(C)** and Flow cytometric analysis of apoptosis **(D)** of sorted NK cells (CD3^−^CD19^−^ NKp46^+^) after *in vitro* stimulation with 50 or 100 ng/ml IL-15 for 5 days from control vs. Foxo1^Δ/Δ^ mice. Each dot represents one mouse. Four and six littermates were included for **(A,B)**, respectively; 2 littermates were included for **(C)** 2 and 4 littermates were included for 50 and 100 ng/ml IL-15 stimulation, respectively, for **(D)**. (Error bars indicate SD; unpaired Student's *t*-test with generalized linear models; *p < 0.05, **p < 0.01, ***p < 0.001, and ****p < 0.0001, control vs. Foxo1^Δ/Δ^ mice).

To find more evidence to support our finding of increased NK cells by hematopoietic-specific deletion of Foxo1, we next explored the proliferation potential and cell survival of NK cells by *in vivo* and *ex vivo* experimental models. In fresh isolated BM cells and splenocytes, we found that the protein level of Ki-67 was significantly increased in total NK cells, especially in CD27^+^ NK cells, including CD27^+^CD11b^−^ (CD27 SP) and CD27^+^CD11b^+^ (DP) NK subsets, of Foxo1^Δ/Δ^ mice (Figure 2A). In fresh isolated BM cells and splenocytes, we also found a moderate increased NK cell apoptosis in Foxo1^Δ/Δ^ mice, indicated by Annexin V and 7-AAD staining (Figure 2B).

To further explore whether the enhanced proliferation potential and increased apoptosis of NK cells by hematopoietic-specific Foxo1 deletion is cell-intrinsic or not, we used NK cells with high purity (>95%) sorted from the splenocytes of both control and Foxo1^Δ/Δ^ mice for IL-15 stimulation. After *in vitro* cultured with 50 or 100 ng/ml IL-15 for 5 days, NK cells derived from Foxo1^Δ/Δ^ mice expanded more than those from control mice (Figure 2C), whereas no difference of cell apoptosis between both mice (Figure 2D).

In all, these data demonstrated that Foxo1 repressed NK cell proliferation in a cell-intrinsic manner. The increased cell apoptosis in fresh isolated BM cells and splenocytes of Foxo1^Δ/Δ^ mice might be caused by other lymphocytes, such as T cell activation and subsequent cytokine secretion (23, 24).

### Hematopoietic-Specific Deletion of Foxo1 Downregulates mRNA Expression of Cell-Cycle Repressors in NK Cells

**Figure 3:**
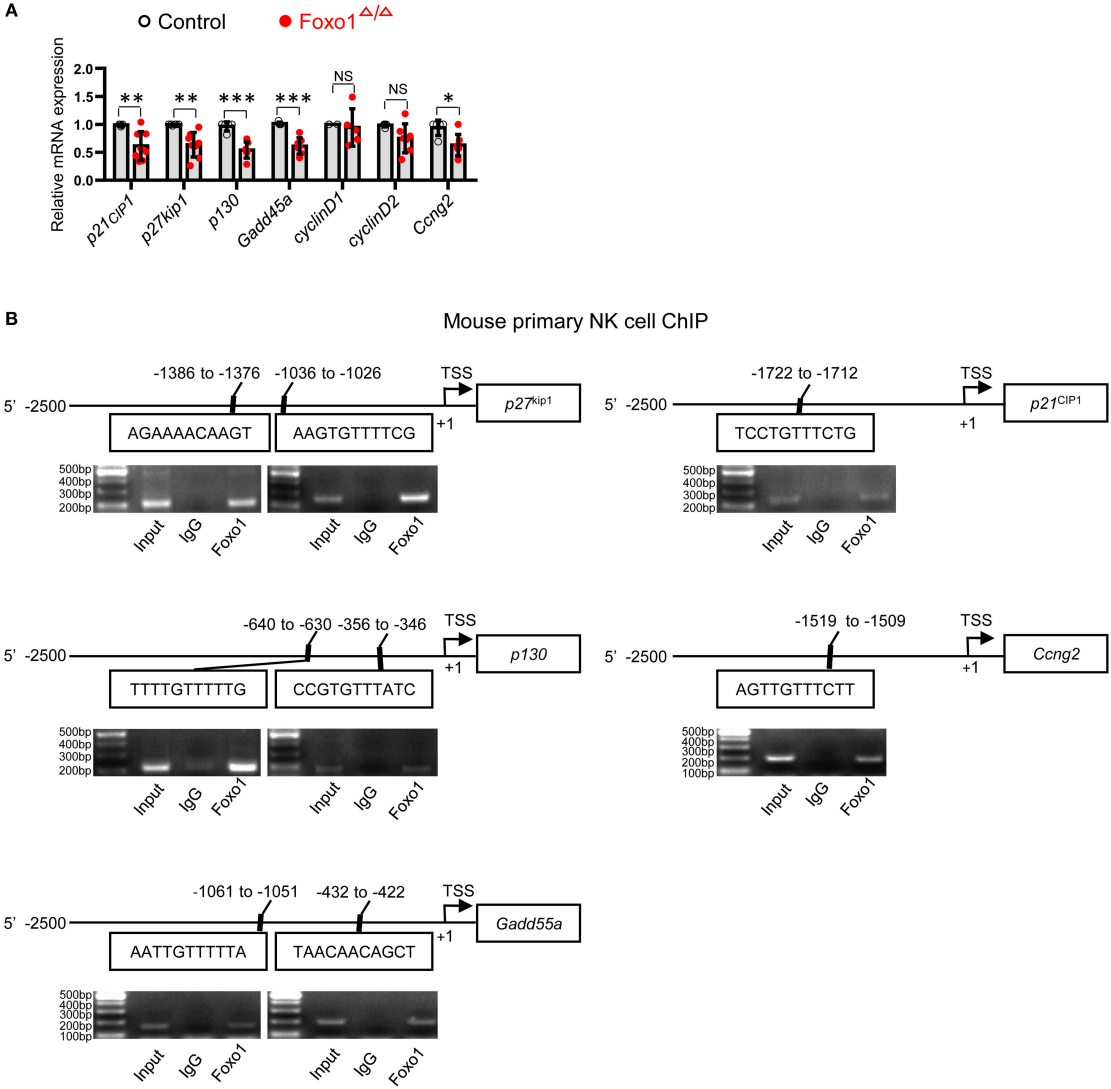
Hematopoietic-specific deletion of Foxo1 downregulates cell-cycle repressors of NK cells. **(A)** q-RT-PCR analysis of direct target genes of Foxo1 responsible for cell cycle in sorted splenic NK cells (CD3^−^CD19^−^NKp46^+^, purity >95%) from control and Foxo1^Δ/Δ^ mice. Each dot represents one mouse. 5 littermates were included for *p21*^*cip*1^; 4 littermates were included for *p27*^kip1^, *Gadd45a, p130, cyclinD2*, and *Ccng2*; and 2 replicates were included for *cyclinD1* (Error bars indicate SD; unpaired Student's *t*-test with generalized linear models; *p < 0.05, **p < 0.01, and ***p < 0.001, control vs. Foxo1^Δ/Δ^ mice). **(B)** Schematic structure of indicated mouse cell-cycle repressors with putative Foxo1 binding sites (Top panels). ChIP assay of Foxo1 binding to the promoter region of indicated cell-cycle repressors in sorted splenic NK cells (CD3^−^CD19^−^NKp46^+^) from wild type mice (Bottom panels). The precipitated DNA by Foxo1 was performed with PCR. Representative PCR gel pictures from 1 of 2 replicates are shown.

To further elucidate the potential mechanism of altered proliferation in Foxo1-deficient NK cells, we next determined the direct target genes of Foxo1 that associated with cell cycle control in Foxo1^Δ/Δ^ mice, including cyclin-dependent kinase inhibitor 1A (*p21*^cip1^), cyclin-dependent kinase inhibitor 1B (*p27*^kip1^), growth arrest and DNA-damage-inducible 45 alpha (*Gadd45a*), RB transcriptional corepressor like 2 (*p130*) and growth-arrest cycilnG2 (*Ccng2*) (25, 26). We observed significant downregulation of *p21*^CIP1^, *p27*^kip1^, *Gadd45a, p130*, and *Ccng2* mRNA levels, all of which are cell-cycle repressors (27–31), in Foxo1^Δ/Δ^ mice (Figure 3A).

As Foxo family members regulate their target genes in a highly cell- and context-specific manner (17, 32, 33), we next try to find more direct evidence supporting Foxo1 regulating the mRNA levels of these above cell-cycle repressors in NK cells. Our Foxo1 ChIP assay by using wildtype NK cells revealed that Foxo1 could directly bind to the promoter region of these cell-cycle repressors: *p21*^cip1^ (−1722 to −1712: TCCTGTTTCTG), *p27*^kip1^ (−1386 to −1376: AGAAAACAAGT; −1036 to −1026: AAGTGTTTTCG), *p130* (−640 to −630: TTTTGTTTTTG; −356 to −346: CCGTGTTTATC), *Gadd45a* (−1061 to −1051: AATTGTTTTTA; −432 to −422: TAACAACAGCT), and *Ccng2* (−1519 to −1509: AGTTGTTTCTT) (Figure 3B).

All these data suggested that the direct binding to the promoter of the cell-cycle repressors by Foxo1 might be responsible for Foxo1 regulating the mRNA expression of these above confirmed cell-cycle repressors in NK cells.

### Hematopoietic-Specific Deletion of Foxo1 Promotes NK Cell Maturation

**Figure 4:**
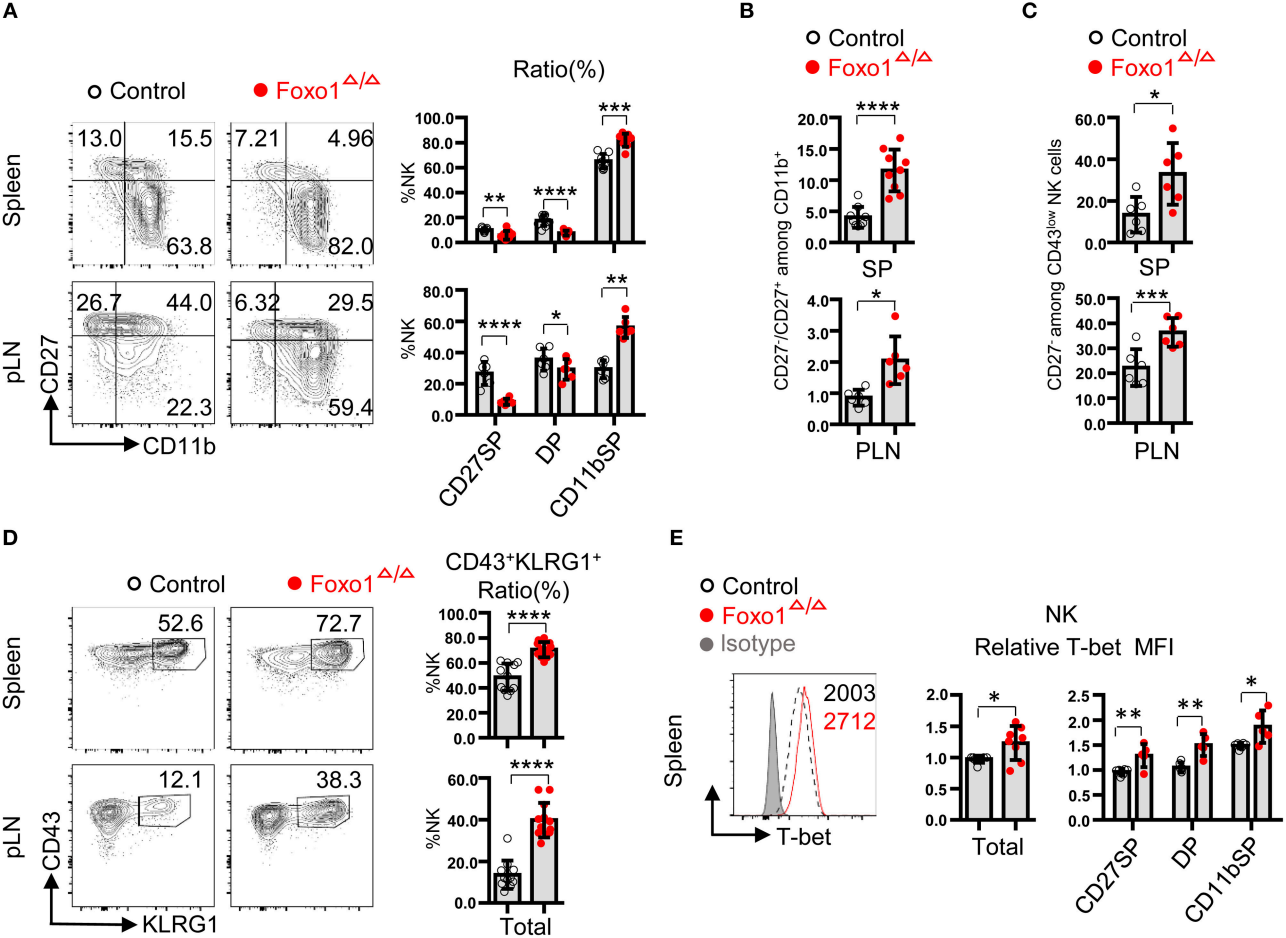
Hematopoietic-specific deletion of Foxo1 promotes NK cell maturation. **(A)** Flow cytometric analysis and frequencies of indicated subsets in NK cells (CD3^−^CD19^−^ NKp46^+^) distinguished by the expression of CD27 and CD11b in the spleen and peripheral lymphoid node (pLN) from control and Foxo1^Δ/Δ^ mice. **(B)** The calculated ratio between CD27^−^ vs. CD27^+^ cells among CD11b^+^ NK cells in both spleen and pLN from control and Foxo1^Δ/Δ^ mice. **(C)** Frequencies of CD27^−^ among CD43^low^ NK cells (CD3^−^CD19^−^NKp46^+^) in the spleen and pLN from control and Foxo1^Δ/Δ^ mice. **(D)** Flow cytometric analysis and frequencies of CD43^+^KLRG1^+^ NK cells (CD3^−^CD19^−^NKp46^+^) in the spleen and pLN from control and Foxo1^Δ/Δ^ mice. **(E)** Intracellular flow cytometric analysis of T-bet expression (MFI relative to littermate control) in NK cells (CD3^−^CD19^−^NKp46^+^) in the spleen from control and Foxo1^Δ/Δ^ mice. Each dot represents one mouse. At least three littermates were included for **(A–E)**. (Error bars indicate SD; unpaired Student's *t*-test with generalized linear models; ^*^p < 0.05, ^**^p < 0.01, ^***^p < 0.001, ^****^p < 0.0001, control vs. Foxo1^Δ/Δ^ mice).

As some of our above data, such as the increased ratio and cell number of NK cells in Foxo1^Δ/Δ^ mice, is inconsistent with the findings from *Foxo1*^fl/fl^/*Ncr1*-iCre mice (named as Foxo1^ΔNK^ mice), we then revisited the NK cell maturation in Foxo1^Δ/Δ^ mice. After the commitment to the NK cell lineage, NK cells undergo three sequential stage of maturation based on the surface expression of CD27 and CD11b: CD27^+^CD11b^−^ NK cells (CD27 single positive, CD27SP), CD27^+^CD11b^+^ (double positive, DP), and CD27^−^CD11b^+^ (CD11b single positive, CD11bSP). During the late stage of maturation, NK cells gradually upregulate CD11b expression, downregulate CD27 expression, and finally obtain co-expression of CD43 and KLRG1 (5, 15, 16, 34). Our data showed that hematopoietic-specific deletion of Foxo1 also resulted in significant increase in the proportion of CD11b SP NK cells, but decreased CD27SP and DP NK cells in the periphery, including spleen and pLN, compared with those in control mice (Figure 4A). Consistently, a specific analysis of CD11b^+^ NK cells revealed a strong bias toward development of CD27^−^ over CD27^+^ cells in the spleen and pLN of Foxo1^Δ/Δ^ mice, compared with those in control mice (Figure 4B). Furthermore, CD43^low^ NK cells from Foxo1^Δ/Δ^ mice showed reduced CD27 expression (Figure 4C). In comparison with control mice, we found a similar increase in the proportion of CD43^+^KLRG1^+^ NK cells in both spleen and pLN of Foxo1^Δ/Δ^ mice (Figure 4D), which is related to NK cell terminal maturation (7, 8). Accordingly, Foxo1 abrogation promoted the expression of T-bet, a master regulator for NK cell development (35), in NK cells in periphery lymphoid organs (Figure 4E).

Altogether, our data indicated that hematopoietic-specific deletion of Foxo1 led to elevated NK cell maturation by NK cells, consistent with our previous study in the murine model of *Ncr1*-iCre-mediated Foxo1 deletion (11).

## Discussion

Previous studies have demonstrated numerous positive and negative transcriptional factors responsible for NK cell development and maturation by using NK cell specific knockout mice, i.e., *Ncr1*-iCre mediated targeted gene deletion. By using this model, we previously found a negative transcriptional factor, Foxo1, for NK cell maturation and effector function (11). However, it could not unmask the role of an interesting transcriptional factor on the specification and early development of NK cells, especially the effect of Foxo1 expressed in HSC, CLP, and NK progenitor cells on later NK cell specification and maturation. In this current study, we generated a hematopoietic cell specific conditional deletion of Foxo1 model by utilizing *Vav1*-iCre mice (12), which could delete Foxo1 expression starting at the stage of HSC to committed NK cells as well as other lymphocytes. By this murine model, we found that Foxo1 deletion by *Vav1*-iCre mice led to increased cell number of CLP, pre-pro NK cells and Lin^−^CD122^+^ cells, indicative of Foxo1 on repressing NK cell specification. As hematopoietic-specific deletion of Foxo1 also affects T and B cell development and function, the current study couldn't exclude whether altered T and B cell development by Foxo1 deletion would also participate in disrupted NK cell specification or not.

Our data showed that hematopoietic-specific deletion of Foxo1 resulted in enhanced maturation of NK cells, including increased percentage of CD11b SP and CD43^+^KLRG1^+^ NK cell subpopulations, in periphery lymphoid organs. Accordingly, Foxo1 abrogation promoted the expression of T-bet in NK cells. This was consistent with our previous reporting by using *Ncr1*-iCre mediated gene deletion model (11). Interestingly, the increment of maturation seems to be more obvious in Foxo1^Δ/Δ^ mice than that in Foxo1 ^*fl*/*fl*^-*Ncr1*-iCre (Foxo1^ΔNK^) mice. For example, the relative immature, CD27 SP, NK cells were significantly decreased in Foxo1^Δ/Δ^ mice but with no statistical significance in Foxo1^ΔNK^ mice, although we did not do the side by side study by using Foxo1^Δ/Δ^ and Foxo1^ΔNK^ mice from the same littermates.

To our surprise, hematopoietic-specific deletion of Foxo1 also resulted in increased cell number and proliferation potential of NK cells in a cell-intrinsic manner, which was inconsistent with the finding by *Ncr1*-iCre mediated Foxo1 deletion. In support of this, we found an obvious increased Ki-67 expression, but a moderate increased apoptosis, in NK cells from Foxo1^Δ/Δ^ mice. We also excluded the extrinsic effects on NK cell proliferation and apoptosis that might be caused by T and B cells via *ex vivo* IL-15 stimulation of purified NK cells. After 5 days of IL-15 stimulation, purified NK cells from Foxo1^Δ/Δ^ mice showed meaningful expanding but with no difference of cell apoptosis in compare with control mice. This finding suggested that Foxo1 repressed NK cell proliferation in a cell-intrinsic manner. However, there was no alteration regarding NK cell proliferation and apoptosis in Foxo1^ΔNK^ mice that indicated by BrdU and Annexin V assay, respectively (Data not shown).

Mechanistically, we found significant reduced expression of cell-cycle inhibitory genes, such as *p21*^CIP1^, *p27*^kip1^, *p130, Gadd45a*, and *Ccng2* in NK cells from Foxo1^Δ/Δ^ mice. Among above repressors, *p21*^CIP1^ and *p27*^kip1^ are conserved target genes of Foxo1 and efficiently arrest cell cycle by encoding critical cyclin-dependent kinase inhibitors. *p21*^CIP1^ inhibits cell cycle transition from G1 to S phase by cyclin binding (36) and repression of DNA replication through binding to *PCNA* promoter (37, 38). *p27*^kip1^ binds to and represses Cyclin E/Cdk2 complexes in G1 phase, which also inhibits transition from G1 to S phase (39). Further ChIP assay also confirmed the direct binding of Foxo1 to the promoter region of the above cell-cycle inhibitors. Thus, our data suggested that the direct binding of Foxo1 on the promoter region of cell-cycle repressor genes might be responsible for Foxo1 repressing NK cell proliferation. Unfortunately, we didn't determine the protein levels of the cell-cycle repressors in Foxo1-deficient NK cells, which was a limitation of our study and need to be further explored.

A recent study suggests a new paradigm that a gene expressed during the ontogeny of NK cell, though lost its expression in mature NK cell, may also affect NK cell development and effector functions (40). Recombination-activating gene (RAG) family proteins, including RAG1 and RAG2, plays critical roles in mediating V(D)J gene rearrangement at the antigen receptor loci during T and B cell development, giving rise to lymphocytes with unique specificity. T and B cells are completely absent, while NK cells are present in normal numbers in RAG-deficient mice (41, 42). Recently, Joseph C. Sun's lab reported that, during the ontogeny of NK cell, highest levels of RAG expression in CLPs from BM, with expression decreasing as NK cells undergo maturation (40). NK cells lack of RAGs expression or RAG endonuclease activity during ontogeny showed a cell-intrinsic hyperresponsiveness but failed to expand and survive following virus infection (40). Mechanism study revealed that a reduced expression of DNA damage response mediators and defects in the repair of DNA breaks in RAGs deficient NK cells were responsible for the changed phenotype in RAG-deficient NK cells (40). Previous study revealed that, in pro-B cells, Foxo1 was involved in regulating DNA damage-induced *RAG*1 and 2 expression by directing binding to the *RAG* enhancer (43). These evidence, combined with our findings, suggested that the relative higher expression of Foxo1 in CLP and early NK precursors may also contribute to the later mature NK cell proliferation and responsiveness, which warrantied another separate study in the future.

Collectively, our study revealed that Foxo1 also acted as a negative checkpoint of NK cell specification and proliferation, which might provide a new strategy to expanding NK cells for immunotherapy based on modulating Foxo1 activity in CLP, NK precursor as well as mature NK cells.

## Ethics Statement

This study was carried out in accordance with the recommendations of the Animal Ethics Committee of the Army Medical University. The protocol was approved by the the Animal Ethics Committee of the Army Medical University (Chongqing, China).

## Author Contributions

PH designed and performed experiments, analyzed data, and wrote the manuscript. FW, YY, WL, MM, SW, HP, LW, and RZ performed the experiments. JY and SI revised the manuscript. BC and XL designed the experiments, analyzed data, and edited the manuscript. YD devised the concept, designed the research, supervised the study, and wrote the paper.

### Conflict of Interest Statement

The authors declare that the research was conducted in the absence of any commercial or financial relationships that could be construed as a potential conflict of interest.
