# Supplementary material for: Hematopoietic-Specific Deletion of Foxo1 Promotes NK Cell Specification and Proliferation
Source: Front Immunol. 2019 May 8;10:1016. doi: 10.3389/fimmu.2019.01016 (PMC6519137; doi:10.3389/fimmu.2019.01016)
Supplement: Supplementary file 1 [file Image_1.pdf]

**Hematopoietic-specific Deletion of Foxo1 Promotes NK Cell Specification and Proliferation**

Pei Huang, Fangjie Wang, Yao Yang, Wenjing Lai, Meng Meng, Shuting Wu, Hongyan Peng, Lili Wang, Rixing Zhan, Saber Imani, Jianhua Yu, Bingbo Chen*, Xiaohui Li* and Youcai Deng*

**Supplemental Materials**

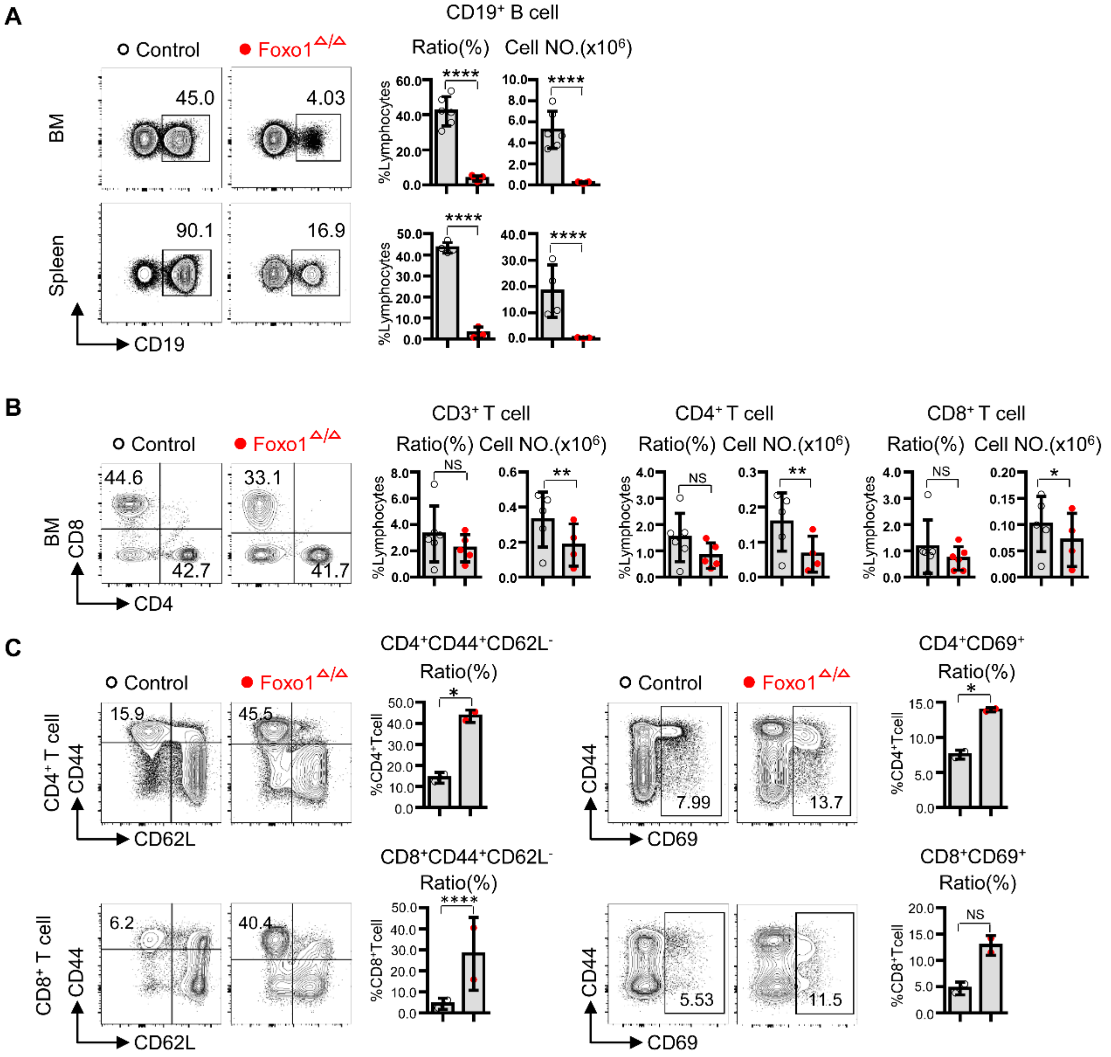


**Supplemental Figure 1 The effect of hematopoietic-specific deletion of Foxo1 on B cells and T cells.** (**A**) Frequency and enumeration of $CD19^{+}$ B cells ($CD3^{-}CD19^{+}NKp46^{-}$) in the BM from control and $Foxo1^{\Delta/\Delta}$ mice. (**B**) Frequency and enumeration of $CD4^{+}$ T cells ($CD3^{+}CD4^{+}CD8^{-}$) and $CD8^{+}$ T cells ($CD3^{+}CD4^{-}CD8^{+}$) in the BM from control and $Foxo1^{\Delta/\Delta}$ mice. (**C**) Frequency of effector T cell: $CD44^{+}CD62L^{-}$ and $CD69^{+}$ in $CD4^{+}$ T cells ($CD3^{+}$ $CD4^{+}$ $CD8^{-}$) and $CD8^{+}$ T cells ($CD3^{+}$ $CD4^{-}$ $CD8^{+}$) in the spleen. Each dot represents one mouse. At least 4 littermates were included for A and B, 2 littermates were included for C (Error bars indicate SD; unpaired Student's *t* test with generalized linear models; $*p < 0.05$, $** \ p < 0.01$, $*** \ p < 0.001$, and $****p < 0.0001$, control versus $Foxo1^{\Delta/\Delta}$ mice).
